# Use of Protein Pegylation to Prolong the Antiviral Effect of IFN Against FMDV

**DOI:** 10.3389/fmicb.2021.668890

**Published:** 2021-05-05

**Authors:** Fayna Diaz-San Segundo, Gisselle N. Medina, Paul Azzinaro, Joseph Gutkoska, Aishwarya Mogulothu, Sarah E. Attreed, Kimberly R. Lombardi, Jacob Shields, Teresa A. Hudock, Teresa de los Santos

**Affiliations:** ^1^Plum Island Animal Disease Center (PIADC), ARS, USDA, Greenport, NY, United States; ^2^Kansas State University College of Veterinary Medicine, Manhattan, KS, United States; ^3^Department of Pathobiology and Veterinary Science, University of Connecticut, Storrs, CT, United States; ^4^ORISE-PIADC Research Participation Program, Oak Ridge, TN, United States; ^5^Elanco Animal Health, Inc., Greenfield, IN, United States

**Keywords:** FMDV, foot-and-mouth disease, type I interferon, IFN, PEGylation, biotherapeutics

## Abstract

Interferons (IFNs) are considered the first line of defense against viral diseases. Due to their ability to modulate immune responses, they have become an attractive therapeutic option to control virus infections. In fact, like many other viruses, foot-and-mouth disease virus (FMDV), the most contagious pathogen of cloven-hoofed animals, is highly sensitive to the action of IFNs. Previous studies demonstrated that type I, II, and III IFNs, expressed using a replication defective human adenovirus 5 (Ad5) vector, can effectively block FMDV replication *in vitro* and can protect animals when challenged 1 day after Ad5-IFN treatment, in some cases providing sterile immunity. Rapidly spreading foot-and-mouth disease (FMD) is currently controlled with vaccination, although development of a protective adaptive immune response takes 5–7 days. Therefore, an optimal strategy to control FMD outbreaks is to block virus replication and spread through sustained IFN activity while the vaccine-stimulated adaptive immune response is developed. Challenges with methods of delivery and/or with the relative short IFN protein half-life *in vivo*, have halted the development of such approach to effectively control FMD in the animal host. One strategy to chemically improve drug pharmacodynamics is the use of pegylation. In this proof-of-concept study, we demonstrate that pegylated recombinant porcine (po)IFNα displays strong and long-lasting antiviral activity against FMDV *in vitro* and *in vivo*, completely protecting swine against FMD for at least five days after a single dose. These results highlight the potential of this biotherapeutics to use in combination with vaccines to fully control FMD in the field.

## Introduction

Foot-and-mouth disease (FMD) is a severe, highly contagious disease of cloven-hoofed animals including ruminants and swine. The disease is characterized by high fever and the appearance of vesicular lesions around the mouth, feet, and teats that resolve in a relative short time of approximately 2 weeks (Grubman and Baxt, 2004). However, ruminants that recover from disease, become asymptomatic virus carriers in large the numbers (Maree et al., 2016; Arzt et al., 2018). The etiologic agent of this disease is the FMD virus (FMDV), a member of the *Aphthovirus* genus within the Picornaviridae family. FMDV consists of an approximately 8.5 kb single-strand RNA of positive polarity surrounded by an icosahedral capsid comprised of 60 copies of four viral encoded structural proteins. Like other RNA viruses, FMDV has an error-prone polymerase that leads to a high mutation rate during genome replication and confers rapid adaptation on the virus. FMDV is antigenically variable and displays seven serotypes including A, O, C, Asia, and South African Territories (SAT) 1, 2, 3, and multiple subtypes (Knowles and Samuel, 2003). Such genetic plasticity and fast rate of replication are probably the drivers of high FMDV morbidity in over 70 species of ungulates.

Foot-and-mouth disease is endemic in at least the following three continents: Africa, Asia, and South America. Recurrent outbreaks in those areas not only affect animal productivity but create substantial trade barriers that impact economic and social development. Current disease control measures include broad surveillance, enforcement of sanitary policy, and vaccination. The current approved FMD vaccine consists of purified chemically inactivated whole-virus (binary ethylenimine -BEI- treated) formulated with oil-based or aluminum adjuvants that is used with a boosting protocol for ensuring long term protection (Doel, 2003). Multiple experimental vaccines have shown promising results and are under advanced stages of development, however, their availability is limited (Diaz-San Segundo et al., 2017; de Los Santos et al., 2018). FMD vaccines are serotype specific and require approximately 7 days to induce a protective immune response, leaving a window of time when the virus could still infect animals in case of an outbreak. To address the need to protect vaccinated animals during this window of susceptibility, we have previously used IFN based therapeutics. Delivery of porcine IFNα/β using a replication deficient adenovirus 5 vector (Ad5-poIFNα), effectively protected swine challenged with FMDV A24 Cruzeiro as early as 1 day post-inoculation (Chinsangaram et al., 2003). In some instances, protection could last for 3–4 days post-Ad5-poIFNα-inoculation (Moraes et al., 2003) and was effective against multiple FMDV serotypes (Dias et al., 2011). Moreover, in proof-of-concept studies Moraes et al. (2003), demonstrated that a combination of Ad5-poIFNα with an Ad5 vaccine that delivers FMDV A24Cru empty capsids (Ad5-FMD-A24) could induce complete protection of swine challenged with FMDV at 1–3 days post-infection (dpi) while a strong adaptive immune response was mounted.

A cost-effective alternative to the use of IFN delivered by an Ad5 vector, is the use of recombinant protein expressed in bacteria or other eukaryotic systems such as yeast or insect cells. Over the last 30 years many such studies have been performed using IFNs to control human pathogens such as Hepatitis C, Hepatitis B, Venezuelan equine encephalitis (VEE), and others (Hoofnagle and Seeff, 2006; Friedman, 2008). However, the use of IFNs requires extensive testing in the species of interest in order to evaluate the metabolic rate and potential adverse systemic effects of individual preparations. In this regard, many approaches to change the pharmacokinetic profiles of IFNs have been examined. These include the covalent modification of IFN with polyethyleneglycol (PEG) molecules (PEGylation) or the expression of recombinant IFN fused to immunoglobulins Fc fragments, albumin or other proteins. These modified-IFNs have been tested for the treatment of multiple human diseases such as hepatitis, multiple sclerosis and cancer (Fried et al., 2002; Woo et al., 2017; Ortiz et al., 2018; Lazear et al., 2019). Use of these new IFN-modified platforms should improve its biotherapeutic function in the animal setting as well.

In this study, we have evaluated the antiviral activity of pegylated poIFNα (PEGpoIFNα) against FMDV in swine. Our results demonstrate that this molecule displays sustained and strong bioactivity in this species, protecting animals against challenge with FMDV for at least 5 days post-treatment. These results highlight the potential use of PEGpoIFNα in combination with vaccines to induce immediate and long-term protection against such a feared pathogen of the agricultural industry.

## Materials and Methods

### Cells and Viruses

Human embryonic kidney (HEK) 293 cells (ATCC CRL-1573) were used to generate and propagate recombinant Ad5s. Baby hamster kidney cells strain 21 (BHK-21, clone 13) (ATCC CCL-10) were used to propagate challenge virus and to measure virus titers in plaque assay. Porcine kidney cells (IB-RS2) were obtained from the Foreign Animal Disease Diagnostic Laboratory (FADDL) at Plum Island Animal Disease Center (PIADC), Greenport, NY and use to measure antiviral activity. MDBK-t2 [Madin-Darby bovine kidney cells transfected with plasmid expressing the human MxA promoter linked to a chloramphenicol acetyltransferase (CAT) reporter and with resistance to blasticidin (Fray et al., 2001)] were kindly provided by B. Charleston (Institute for Animal Health, Pirbright, United Kingdom). All cells were maintained in Eagle’s minimal essential medium (EMEM) containing either 10% calf serum or 10% fetal bovine serum (FBS) supplemented with antibiotics, glutamine, and non-essential amino acids. MDBK-t2 media was also supplemented with 10 μg/ml blasticidin (Invitrogen. Carlsbad, CA, United States). Replication-defective recombinant Ad5s containing poIFNα were produced as previously described (Moraes et al., 2003; Diaz-San Segundo et al., 2011).

The challenge virus FMDV A24 Cruzeiro/55 (A24Cru) was obtained from vesicular fluid of infected swine, followed by titering in swine and in tissue culture, and stored in aliquots at −70°C.

PEGylated porcine IFNα (PEGpoIFNα) was provided by Elanco Animal Health, Inc.

### FMDV Cell Infections

Cultured cell monolayers were infected with FMDV at indicated multiplicity of infection (moi). After 1 h adsorption at 37°C, unabsorbed virus was removed by washing the cells with a solution containing 150 mM NaCl in 20 mM morpholine ethanesulfonic acid (MES) pH = 5.5, before adding minimal essential medium (MEM) and proceeding with incubation at 37°C in 5% CO_2_. Infected cells were frozen at 1, 3, 6, and 24 h and virus titers were determined after thawing, by plaque assay on BHK-21 cells. Plaques were counted at 48 h post-inoculation (hpi).

### *In vitro* Anti-FMDV Antiviral Bioassay

Porcine IB-RS2 cells were treated with twofold serial dilutions of Ad5-expressed poIFNα or PEGpoIFNα (starting material: 2^–1^ to 2^–12^ dilutions, or ∼10 μg/ml, respectively) and incubated for up to 7 days. At 1 day intervals, supernatants were collected from individual plates and stored at −70°C. Frozen supernatants samples containing Ad5-expressed poIFNα or PEGpoIFNα were thawed, serial diluted and applied to fresh IB-RS2 cell monolayers in 96 well plates using ten replicates per IFN dilution. Treated cells were incubated for 24 h at 37°C, followed by removal of the IFN containing supernatant, and infection with FMDV at a MOI 0.1. After incubation for 72 h at 37°C with 5% CO2, cell viability was measured using an MTT assay (Ramanathan et al., 2015). OD values of one represent 100% viability, or absence of cytotoxicity. OD values of 0.04 represent maximum cytotoxicity.

### Animal Experiments

The pharmacokinetic study was performed at Blue River Research Services (Carthage, IN, United States), and was conducted in compliance with BRRS ACUP No. S-01. Efficacy study was performed in the high-containment facilities of the Plum Island Animal Disease Center, conducted in compliance with the Animal Welfare act (AWA) (2011) Guide for Care and Use of Laboratory Animals, 2002 PHS Policy for the Humane Care and Use of Laboratory Animals, and United States. Government Principles for Utilization and Care of Vertebrates Animal Used in Testing, Research and Training (IRAC, 1985), as well as specific animal protocols reviewed and approved by the Institutional Animal Care and Use Committee (IACUC) of the Plum Island Animal Disease Center (USDA/APHIS/AC Certificate number: 21-F-0001; Protocol 151-19R).

#### Pharmacokinetics Animal Study

Eighteen pigs (nine castrate and nine gilt, weight between 7.92–9.81 kg) were randomly assigned to one of three treatment groups (3M/3F per group) and acclimated for 5 days. Animals were group housed by treatment and gender (three animals per pen), in indoor pens with *ad libitum* access to unmedicated feed and water. Group 1 received a single administration of 25 μg/kg of PEGpoIFNα intravenously (IV); group 2 received a single administration of 25 μg/kg of PEGpoIFNα intramuscularly (IM); and group 3 received a single administration of 50 μg/kg of PEGpoIFNα IM. Animals were administered test article according to the assigned treatment group in the neck using a 5 ml syringe, with a 20G × 1 inch needle.

Blood samples (approximately 1–2 ml) were collected from each animal the day before dosing (*t* = 0), and at 30 min, 1, 2, 4, 8, 12, 24, 36, 48, 60, 72, 84, 96, 108, 120, 144, 168, 192, 216, and 240 h post-dose administration. Blood samples were collected in a sodium citrate tube *via* jugular venipuncture within 10% of the schedule time for time points <4 h post-administration, within 30 min for time points between 4 and 24 h and within 2 h for time points greater than 24 h. Blood samples were separated by centrifugation and stored at approximately −70°C. Resultant plasma samples were shipped to AIT BioSciences (Indianapolis, IN, United States) for PEGpoIFNα analysis.

#### Efficacy Animal Study

Sixteen Yorkshire gilts or castrated males (5 weeks old and weighing approximately 18–23 kg each) were randomly divided in four groups of four animals each and were acclimated for 1 week. Group 1 received 200 μg/kg of PEGpoIFNα IM 1 day before challenge; group 2 received 200 μg/kg of PEGpoIFNα IM 5 days before challenge; group 3 was treated with 10^10^ plaque forming units (PFU)/animal of Ad5-poIFNα subcutaneous (SQ) 5 days before the challenge; group 4 was inoculated with PBS SQ 1 day before the challenge and was considered as the negative control the negative control group. All animals were challenged intradermally (ID) in the rear heel bulb with 100 tissue culture infectious dose 50 (TCID_50_) of FMDV A24Cru at indicated times post-treatment. This dose had been previously validated as consistently causing full disease at 3–5 days post challenge (Ramirez-Carvajal et al., 2016).

Serum and heparinized blood were collected starting at day 0 for baseline and every day after treatment to examine systemic antiviral activity. Samples of serum and nasal swabs were collected the day of challenge and daily for 7 days after challenge (dpc) and at 14 dpc.

Following challenge, clinical scores were evaluated daily for 7 days by determining the number of toes displaying FMD lesions and the presence of lesions in the snout and/or mouth. The maximum score considered was 17, and lesions restricted to the site of inoculation were not counted. The% of lymphocytes in the white cell population from whole blood collected in EDTA was measured for the first 7 days using a Hemavet cell counter (Drew Scientific-Erba Diagnostics, Miami Lakes, FL, United States).

### Detection of Virus in Sera and Nasal Swabs

Swine sera and nasal swabs were examined for the presence of virus by plaque assays on BHK-21 cells. Virus titers were expressed as log_10_ pfu/ml of serum or nasal swab secretions. The minimal detection level for this assay is 5 pfu/ml. In addition, FMDV RNA was detected by real-time RT-PCR (rRT-PCR) as previously described (Alejo et al., 2013). Cycle threshold (Ct) values were converted to RNA copies per milliliter using the equation derived from analysis of serial 10-fold dilutions of *in vitro* synthesized FMDV RNA of known concentration and expressed as the genome copy number per ml of serum or nasal swab.

### Antiviral Biological Assay in Serum

Antiviral activity was evaluated in serum as previously described (Moraes et al., 2003; de Avila Botton et al., 2006). In brief, serum samples were diluted, and applied to IB-RS2 cell monolayers; after 24 h supernatants were removed, and the cells were infected for 1 h with approximately 100 PFU of FMDV serotype A12 followed by addition of an overlay containing 20% gum tragacanth in tissue culture media. Plaques were observed 24 h later by staining live cells with crystal violet. Antiviral activity (U/ml) was reported as the reciprocal of the highest supernatant dilution that resulted in a 50% reduction in the number of plaques relative to the number of plaques detected in the mock-treated infected cells.

We also used a MxCAT ELISA to determine units of antiviral activity using MDBK-t2 cells as previously described (Diaz-San Segundo et al., 2016). Briefly, cells were seeded into 24 well tissue culture plates at 2 × 10^5^ cells/well and after 24 h incubation at 37°C with 5% CO_2,_ the culture medium was replaced with 0.25 ml of media containing 0.1 ml of either serum samples or nasal swab secretions. Known amounts (from 1.95 to 1,000 U/ml) of recombinant human IFNα2A (PBL Interferon Source, Piscataway, NJ, United States) were used to determine a standard curve. Twenty-four hours after incubation, cells were lysed for 30 min in lysis buffer^®^ and CAT expression was determined using a commercially available CAT-ELISA kit (Roche Applied Sciences, Indianapolis, IN, United States) in accordance with the manufacturer’s protocol. Units of antiviral activity per ml were calculated from the human IFNα2A standard curve.

### Pegylated Porcine-IFNα Detection in Plasma

Presence of PEGpoIFNα in plasma was detected using an ELISA based analytical method that employs an IgM anti-PEG antibody for capture and a biotinylated mouse anti-porcine IFNα antibody for detection. Briefly, plates were coated with anti-PEG capture (Academia Sinica, Taipei, Taiwan) antibody overnight at 2–8°C. Plate was blocked with casein in PBS for 1 h at room temperature while shaking. Plasma samples 1:50 diluted in blocking buffer were incubated overnight at 2–8°C. Biotinylated mouse anti-porcine IFNα antibody (PBI, Piscataway, NJ, United States, biotinylation performed at AIT Bioscience) was then added to the plate and incubated for 1 h at room temperature while shaking at 150 rpm. Signal was detected using Streptavidin-Sulfo Tag (Meso Scale Diagnostics, Rockville, MD, United States) and 1:4 diluted MSD read buffer read on an MSD 6000 imager. Throughout the assay, plates were washed with 1x KPL Wash Buffer (KPL-VWR, Bridgeport, NJ, United States).

After fitting the standard curve of PEGpoIFNα a 4-parameter, 1/y2 weighted logistic model, plasma amounts of PEGpoIFNα were estimated in test samples by extrapolation using a standard curve of pegylated porcine-IFNα calibrators ranging from 3.15 to 1,000 ng/ml.

### Analysis of IFN Stimulated Genes in PBMCs

IFN Stimulated Genes (ISGs) expression in peripheral blood mononuclear cells (PBMCs) was analyzed by quantitative real-time reverse transcription PCR (RT-qPCR) as previously described (Ramirez-Carvajal et al., 2016). Briefly, total RNA was isolated from approximately 10^7^ PBMCs using an RNAeasy extraction kit (Qiagen, Valencia, CA, United States) followed by cDNA synthesis using random hexamers with qScript kit mix (Quanta Biosciences, Gaithersburg, MD, United States). cDNA was used as the template for RT-qPCR with PerfeCTa SYBR green FastMix (Quanta Biosciences, Gaithersburg, MD, United States). Samples were run in an AB 7500 system (Applied Biosystems, Carlsbad, CA, United States). Relative quantification was performed on a panel of ISGs as previously described (Diaz-San Segundo et al., 2010). The expression of each gene of interest was normalized using GAPDH (glyceraldehyde-3-phosphate dehydrogenase). Data was analyzed using the comparative threshold cycle (ΔΔC_*T*_) method relative to baseline levels detected prior to treatment (Livak and Schmittgen, 2001).

### Evaluation of Humoral Immune Response

Neutralizing antibody titers were determined in mice or swine sera samples by end-point titration according to the method of Kärber (OIE, 2018). Antibody titers were expressed as the log_10_ value of the reciprocal of the dilution that neutralized 100 TCID_50_ in 50% of the wells (Diaz-San Segundo et al., 2010).

### Data Analyses

All statistical analysis was conducted using SAS software (Version 9.4, SAS System for Windows, SAS Institute Inc., Cary, NC, United States). All hypotheses were tested at a 2-sided 5% level of significance unless otherwise stated.

All pharmacokinetic analysis was performed using non-compartmental analysis in Phoenix^®^, version 6.3.0.395 (Pharsight Corporation, Mountain View, CA, United States).

Shed RNA viral copies (copy number/ml), nasal swab viral shedding (TCID50/ml), RNA viremia viral copies (copy number/ml), lymphocyte (%), TCID blood virus isolation (TCID50/ml), Vesicular Lesion Score were analyzed by a generalized linear mixed model (GLMM) analysis, with Poisson distribution, log link, fixed effects (Treatment and Treatment × Time) and random repeated measure effect. The covariance structure for the repeated measures effect was selected from the following candidates: compound symmetry (CS), spatial exponential SP(EXP), and spatial spherical SP(SPH). The minimum corrected Akaike Information Criterion (AICC) was used to select the SP(EXP) covariance structure.

MxCAT Elisa (IFN U/ml), Antiviral biologic activity (U/ml), Serum neutralization titers [log10(TCID50/ml)], Weight (Kg), Temperature (F) were analyzed by a linear mixed model (LMM) analysis, with fixed effects (Treatment and Treatment × Time) and random repeated measure effect. The covariance structure for the repeated measures effect was selected from the following candidates: CS, SP(EXP), and SP(SPH). The minimum corrected AICC was used to select the SP(EXP) covariance structure.

## Results

### Pegylation of poIFNα Prolongs Antiviral Activity Against FMDV *in vitro*

**FIGURE 1:**
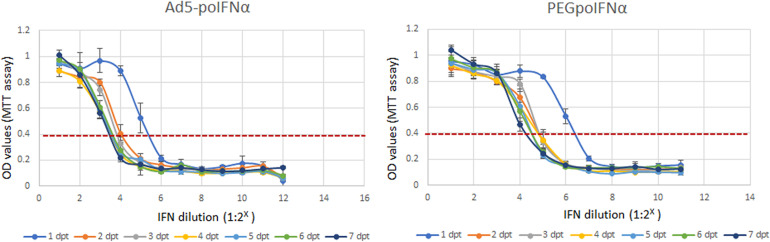
*In vitro* antiviral activity of PEGpoIFNα. Porcine IB-RS2 cells were treated with two-fold serial dilutions of Ad5-poIFNα expressed poIFNα or PEGpoIFNα (starting material 2^– 1^ to 2^– 12^ dilutions or ∼10 μg/ml, respectively), and incubated for up to 7 days post-treatment (dpt). At 1 day intervals, supernatants were collected from individual plates and stored at –70°C. Fresh IB-RS2 cells were treated with the stored supernatants samples containing Ad5-poIFNα expressed poIFNα or PEGpoIFNα. Twenty-four hours post-treatment, IFN containing supernatants were removed and cells were infected with FMDV at MOI-0.1. Cell viability after challenge was measured by MTT assay, 72 h post-infection. OD values of one represent 100% viability, or absence of cytotoxicity. OD values of 0.4 represent maximum cytotoxicity. Results represent the mean of four independent experiments ± SD.

We have previously demonstrated that poIFNα expressed by using either, a replication defective human adenovirus 5 (Ad5-poIFNα) (Chinsangaram et al., 2003; Moraes et al., 2003, 2007) or a VEE virus replicon particle (VRP-poIFNα) (Diaz-San Segundo et al., 2013), is very effective in blocking FMDV replication *in vitro*, and that protection can last for up to 5 days after treatment. To test the effectiveness of the pegylated porcine IFNα (PEGpoIFNα) in comparison to Ad5-poIFNα, we treated porcine IB-RS2 cells with two-fold serial dilutions of Ad5- expressed poIFNα or recombinant PEGpoIFNα (starting material: 2^–1^ to 2^–12^ dilutions, or ∼10 μg/ml, respectively) and incubated the cultures for up to 7 days (Figure 1). By 1 dpt equivalent antiviral activities were detected for both, Ad5-expressed and PEG-poIFNα proteins. However, while the antiviral activity of Ad5-expressed poIFNα waned by 3–4 dpt, a more sustained response was detected in cells treated with PEGpoIFNα which decreased by 4–6 dpt (Figure 1). These results suggest that pegylation of poIFNα does not interfere with its biological activity. Furthermore, the antiviral effect induced by PEGpoIFNα lasts longer as compared to the activity triggered by Ad5- delivered poIFNα.

### Sustained Systemic Detection of poIFNα in Swine After a Single Inoculation of PEGpoIFNα

Pegylation is aimed at increasing protein’s half-life in blood. In order to test if PEGpoIFNα presence in plasma was sustained and for how long, we performed a preliminary pharmacokinetics study in swine. Three groups of six animals were inoculated with 25 μg/kg IV, 25 μg/kg IM or 50 μg/kg IM are were sampled up to 240 h-post inoculation (hpi) (Table 1). Levels of PEGpoIFNα were measured in plasma at indicated time-points in four of the six animals from each treatment group. Animals inoculated IV showed rapid presence of PEGpoIFNα in plasma, with highest levels starting 30 min after inoculation with a harmonic mean half-life (t_1/2_) of 59.7 h (Table 2). As expected, animals inoculated IM took longer time to achieve maximum concentration [T_*max*_ (Table 3)] and never achieved equivalent maximum IFN levels as IV inoculated animals, even when inoculated with double the dose (Table 1). However, half-life was similar regardless the route of inoculation. Furthermore by 72 h, the IFN levels in plasma were equivalent regardless the route of inoculation, showing dose dependance and continued declining until 240 h (Table 1).

**TABLE 1:** Individual and mean plasma concentration (μg/ml) of PEGpoIFNα.

Treatment group	Animal ID	Time__nominal (h)											
		0	0.5	1	4	12	24	72	96	120	168	216	240
TG01 25 μg/kg IV	1	BLQ*	0.281	0.258	0.223	0.174	0.134	0.077	0.048	0.029	0.021	0.013	0.009
	2	BLQ	0.275	0.27	0.232	0.151	0.122	0.067	0.047	0.029	0.019	0.012	0.009
	3	BLQ	0.213	0.195	0.289	0.14	0.099	0.054	0.043	0.024	0.013	0.008	0.005
	4	BLQ	0.242	0.259	0.205	0.153	0.113	0.054	0.042	0.032	0.016	0.009	BLQ
	*N*	–	4	4	4	4	4	4	4	4	4	4	3
	Mean	–	0.253	0.246	0.212	0.155	0.117	0.063	0.045	0.029	0.017	0.01	0.008
	SD	–	0.032	0.034	0.019	0.014	0.015	0.011	0.003	0.004	0.004	0.002	0.002
TG02 25 μg/kg IM	5	BLQ	0.031	0.051	0.087	0.064	0.051	0.039	0.038	0.029	0.015	0.007	BLQ
	6	BLQ	BLQ	0.006	0.029	0.053	0.076	0.063	0.056	0.046	0.024	0.011	0.009
	7	BLQ	0.007	0.015	0.034	0.061	0.064	0.083	0.058	0.036	0.018	0.009	0.006
	8	BLQ	0.014	0.035	0.018	0.087	0.062	0.051	0.043	0.032	0.021	0.011	0.008
	*N*	–	3	4	4	4	4	4	4	4	4	4	3
	Mean	–	0.017	0.027	0.058	0.066	0.063	0.059	0.049	0.036	0.02	0.01	0.008
	SD	–	0.012	0.02	0.031	0.015	0.01	0.019	0.01	0.007	0.004	0.002	0.002
TG03 50 μg/kg IM	9	BLQ	0.046	0.072	0.202	0.196	0.195	0.134	0.122	0.029	0.051	0.029	0.011
	10	BLQ	0.008	0.04	0.128	0.135	0.193	0.135	0.108	0.068	0.058	0.021	0.019
	11	BLQ	0.039	0.094	0.079	0.132	0.124	0.111	0.092	0.079	0.046	0.018	0.015
	12	BLQ	0.007	0.023	0.109	0.139	0.095	0.135	0.103	0.057	0.041	0.028	0.025
	*N*	–	4	4	4	4	4	4	4	4	4	4	4
	Mean	–	0.025	0.057	0.13	0.151	0.152	0.129	0.106	0.058	0.049	0.024	0.017
	SD	–	0.02	0.032	0.052	0.03	0.05	0.012	0.012	0.022	0.007	0.005	0.006

*BLQ, below limit of quantification.

**TABLE 2:** Individual and mean pharmacokinetics parameters for PEGpoIFNα in swine plasma following intravenous administration.

Treatment group	Animal ID	l_*z*_ (1/h)	t_1/2_ (h)	C_0_ (μg/ml)	AUC_*last*_ (h*μg/ml)	AUC_8_ (h*μg/ml)	AUC%Extrap (%)	V_*z*_ (ml/kg)	Cl (ml/h/kg)
TG01 25 μg/kg IV	1	0.0115	60.5	0.306	14	14.84	5.52	147	1.68
	2	0.0097	71.6	0.28	12.9	13.89	6.95	186	1.8
	3	0.0128	54.1	0.233	10.5	10.91	3.63	179	2.29
	4	0.0126	55.1	0.242	11.5	12.28	4.98	162	2.04
	*N*	4	4	4	4	4	4	4	4
	Mean	0.0116	59.7	0.265	12.3	13.0	5.52	168	1.95
	SD	0.00143	8.04	0.0341	1.54	1.74	1.39	17.5	0.269

**TABLE 3:** Individual and mean pharmacokinetics parameters for PEGpoIFNα in swine plasma following intramuscular administration.

Treatment group	Animal ID	l_*z*_ (1/h)	t_1/2_ (h)	T_*max*_ (h)	C_*max*_ (μg/ml)	AUC_*last*_ (h*μg/ml)	AUC_8_ (h*μg/ml)	AUC%Extrap (%)	AUMC_8_ (h*h*μg/ml)	MRT (h)	*F*
TG02: 25 μg/kg IM	5	0.015	46.2	4	0.0868	6.9	7.34	6.03	643	87.6	
	6	0.0126	55.1	24	0.0759	9.8	10.5	7.1	1,100	104	
	7	0.0154	45.2	72	0.0834	9.58	9.97	3.89	912	91.5	
	8	0.0141	49	12	0.087	8.71	9.26	5.88	883	95.3	72%
	*N*	4	4	4	4	4	4	4	4	4	
	Mean	0.0143	48.5	28	0.0833	8.75	9.28	5.73	885	94.7	
	SD	0.00123	4.45	30	0.00519	1.32	1.4	1.34	188	7.2	
TG03: 50 μg/kg IM	9	0.0236	29.4	4	0.202	21	21.4	2.1	1,690	79	
	10	0.0121	57.4	24	0.193	21.3	22.9	7.11	2,270	99.1	
	11	0.0131	52.8	12	0.132	17.5	18.7	6.12	1,860	99.7	
	12	0.0073	95.3	12	0.139	17.3	20.7	16.3	2,800	135	82%
	*N*	4	4	4	4	4	4	4	4	4	
	Mean	0.014	49.5	13	0.167	19.3	20.9	7.9	2,160	103	
	SD	0.00688	27.3	8	0.0361	2.15	1.77	5.99	493	23.4	

### PEGpoIFNα Induces Sustained Systemic Antiviral Activity and Stimulation of ISGs in Swine

**FIGURE 2:**
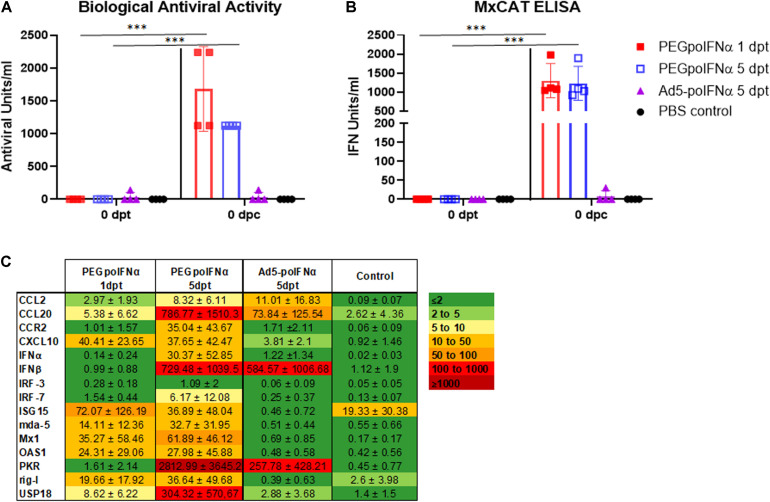
Systemic antiviral response induced by treatment. Bioactivity against FMDV assayed by inhibition of virus growth **(A)** or by MxCAT ELISA **(B)** was determined in sera of treated animals with PEGpoIFNα or with Ad5-poIFNα after 1- or 5-days post-treatment (dpt) right before the animals were challenged (0 dpc). A group of PBS-treated animals was included as a negative control. For all groups, samples taken before any treatment (0 dpt) were used as baseline. Graph represents group average (bar) and individual values of each animal in the group at indicated time points. ^∗∗∗^*P* ≤ 0.001. **(C)** Gene expression in PBMCs of treated animals with PEGpoIFNα or with Ad5-poIFNα after 1 or 5 dpt [right before the animals were challenged (0 dpc)] was measured by qRT-PCR. A group of PBS-treated animals was included as negative control. Results are expressed as relative fold induction values with respect to 0 dpt (ΔΔCT). GAPDH expression was used as normalizer.

Based on the results of PEGpoIFNα pharmacokinetics-animal study, and previous data from animals inoculated with Ad5-poIFNα, we decided to inoculate animals IM with 200 μg/kg of PEGpoIFNα. The selection of dose was based on our previous experience with Ad5-poIFNα and the knowledge on the levels of systemic antiviral activity required for protection in swine (Dias et al., 2011). For comparison, we also inoculated a group of animals with 10^10^ pfu/animal of Ad5-poIFNα. Animals were challenged at 1- or 5- dpt. Systemic antiviral activity was evaluated by measuring inhibition of FMDV growth (biological activity) or by performing an MxCAT ELISA, in the sera obtained from animals prior to treatment (0 dpt, baseline), and at 1 or 5 dpt prior to challenge with infectious FMDV (0 dpc) (Figure 2). One day after treatment, all animals inoculated with PEGpoIFNα showed statistically significant levels of systemic antiviral activity in all assays used for detection. Interestingly, the levels of antiviral activity of PEGpoIFNα treated animals were sustained by 5 dpt, while no antiviral activity could be detected in animals treated with Ad5-poIFNα at the same time (Figure 2).

We also analyzed by real-time RT-qPCR a panel of ISGs that have been previously used in our lab to demonstrate the systemic effect of Ad5-poIFNα on PBMCs (Diaz-San Segundo et al., 2010). ISG expression was measured in PBMCs collected at 1 and 5 dpt with PEGpoIFNα or Ad5-poIFNα. The panel included IFNα and IFNβ, and a small list of well characterized ISGs described in INTERFEROME [a database dedicated to chronicling all genes significantly regulated by IFN (Rusinova et al., 2013)] including IFN-Induced GTP-Binding Protein Mx1, double-stranded RNA-activated protein kinase (PKR), 2′,5′-Oligoisoadenylate Synthetase-Dependent (OAS1), IFN regulatory factor (IRF) -3 and -7, C-C Motif Chemokine Ligand 2 (CCL2), C-C Motif Chemokine Ligand 20 (CCL20), C-X-C Motif Chemokine Ligand 10 (CXCL10), C-C Motif Chemokine Receptor 2 (CCR2), IFN-Stimulated Protein, 15 KDa (ISG15), Ubiquitin Specific Peptidase 18 (UPS18), and also pattern recognition receptors retinoic acid-inducible gene I (rig-I) and melanoma differentiation-associated protein 5 (mda-5).

As observed in Figure 2C, nine out of 15 analyzed genes were upregulated 1 day after PEGpoIFNα inoculation. Interestingly, all genes except for IRF-3 were upregulated by 5 days after PEGpoIFNα treatment. Some of the analyzed genes, including IFNβ, PKR, USP18 and CCL20 were highly upregulated (>300-fold induction). On the contrary, only six out of 15 genes were upregulated in animals treated with Ad5-poIFNα by 5 dpt, and levels of upregulation were low except for IFNβ and PKR which showed similar levels as those detected in PEGpoIFNα treated animals at equivalent times. These results indicate that PEGpoIFNα successfully induced several ISGs and that induction was sustained in time after treatment. Similarly, these data indicate that PEGpoIFNα induces a robust antiviral state detectable at the time of FMDV challenge.

### Treatment With PEGpoIFNα Prolongs Protection Against Challenge With FMDV in Swine

**FIGURE 3:**
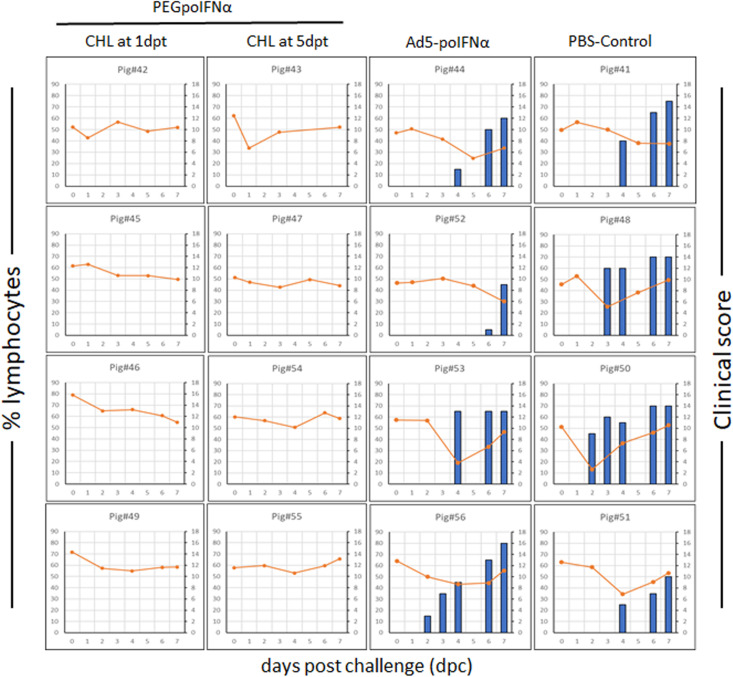
Clinical outcome in animals after challenged with FMDV A24Cru. 18–23 kg castrated male Yorkshire swine (*n* = 4/group) were clinically monitored for 7 days after challenge intradermally (ID) in the heel bulb with FMDV A24Cru and samples of heparinized blood were collected daily. Clinical score (blue bars) and % of lymphocytes (orange line) are represented for each animal individually.

We have previously demonstrated that antiviral activity induced by treatment with Ad5-poIFNα is potentially protective when detected in serum at levels ≥800 U/ml (Dias et al., 2011). Based on those results we predicted that such levels of antiviral activity could protect swine against FMD if elicited by PEGpoIFNα in treated animals. To test our hypothesis, we challenged all animals, including our negative control group with a dose of FMDV A24Cru sufficient to clearly detect disease by 2 dpc (Ramirez-Carvajal et al., 2016). As expected, animals in the PBS control group started showing clinical signs of disease at 2 dpc, with all animals reaching high clinical scores (10–15) (Figure 3). Also, as previously described (Bautista et al., 2003; Diaz-San Segundo et al., 2006), a transient lymphopenia was developed in all animals of the PBS control group that clearly showed clinical disease. Similarly, animals treated with Ad5-poIFNα and challenged at 5 dpt showed vesicular disease and lymphopenia comparable to the PBS control group, with no statistically significant difference between the two groups (data not shown). Remarkably, none of the animals treated with PEGpoIFNα either 1 or 5 days prior to challenge, developed vesicular disease or lymphopenia at any time post-virus challenge. In fact, statistical analysis revealed that, independent of individual variation in clinical signs or in the peak of lymphopenia, a statistically significant difference (*P* ≤ 0.0001) was detected between animals inoculated with PEGpoIFNα and those inoculated either Ad5-poIFNα or PBS control for both parameters.

**FIGURE 4:**
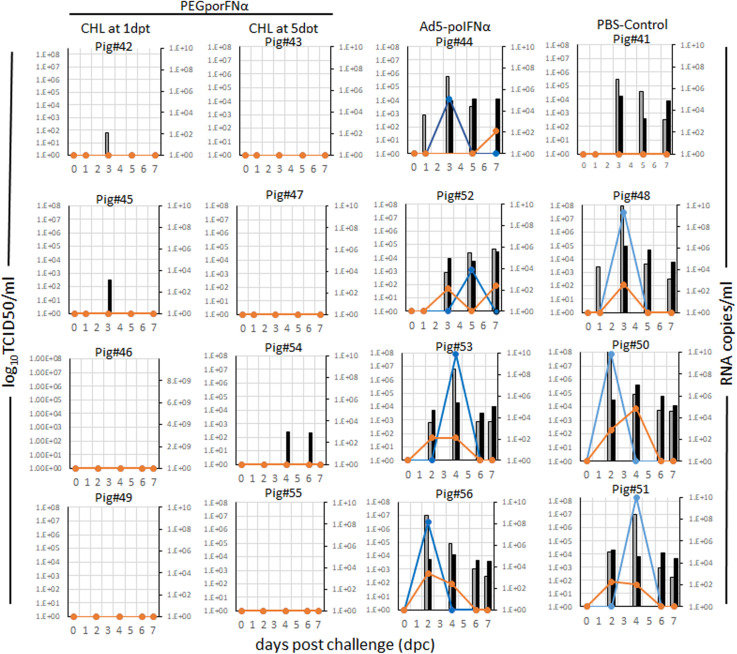
Virus detection in serum and nasal swabs in animals inoculated with different viruses at indicated doses. 18–23 kg castrated male Yorkshire swine (*n* = 4/group) challenged with FMDV A24Cru after indicated treatments (groups) were sampled for 7 days. The amount of virus was detected by virus isolation in serum (orange line) and nasal secretions (blue line) and by qPCR and expressed as RNA copy numbers per ml of serum (gray bars) or per ml of nasal secretions (black bars) are represented for each animal individually.

Parallel to the development of disease, all animals in the PBS control group and animals treated with Ad5-poIFNα, that were challenged at 5 dpt, showed detectable viral RNA in serum and nasal swabs. Additionally, infectious virus was isolated from both, serum and nasal swabs, except for one animal from the PBS control group (Figure 4). On the other hand, infectious virus could not be isolated from any of the animals treated with PEGpoIFNα, and viral RNA was only detected in one out of eight, and two out of eight, sera and nasal swabs samples, respectively (Figure 4). As described for clinical signs and lymphopenia, statistically significant differences for viremia and virus shedding were detected between the two groups treated with PEGpoIFNα when compared to either the PBS control or Ad5-poIFNα treated groups (*P* ≤ 0.0001).

These results indicate that treatment with PEGpoIFNα is effective against FMDV replication and shedding. Furthermore, protection is prolonged in time as compared to treatment with Ad5-poIFNα.

### Development of Adaptive Immune Response After Challenge With FMDV

**FIGURE 5:**
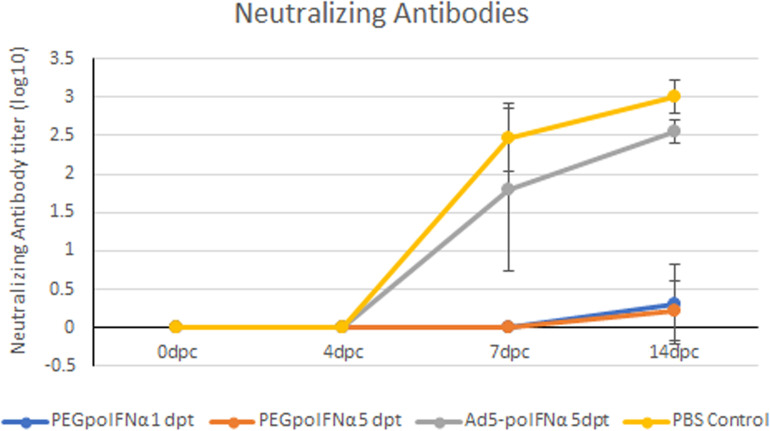
Determination of FMDV neutralizing antibodies. The presence of FMDV neutralizing antibodies was evaluated in sera of animals treated with PEGpoIFNα or with Ad5-poIFNα and challenged after 1 or 5 dpt, by a microtiter neutralization assay on BHK-21 cells. Titers are reported as the log_10_ of the reciprocal of the highest dilution of serum that neutralized the virus in 50% of the wells.

It is well established that FMDV infection induces the development of a strong neutralizing antibody response that ultimately clears viremia (Alexandersen et al., 2003), although some species, like for example cattle, can become persistently infected with virus detected in upper respiratory track tissue for months to years post infection. Also, we have previously reported that swine treated with Ad5-poIFNα could be sterile protected (no clinical FMD, no viremia, undetectable levels of neutralizing antibodies and no detection of antibodies against NS proteins) (Dias et al., 2011). Consistent with those results, in this study we observed that only animals in the PBS control group or treated with Ad5-poIFNα, all challenged at 5 dpt, developed clinical signs, and a strong anti FMDV neutralizing antibody response starting at 7 dpc with no statistically significant differences between these two groups (Figure 5).

## Discussion

It is well established that FMDV is sensitive to IFNs (Medina et al., 2020b). Furthermore, we and others have successfully used IFNs expressed using a replication defective human adenovirus to rapidly block FMDV infection in swine (Chinsangaram et al., 2003; Moraes et al., 2003, 2007; Dias et al., 2011; Kim et al., 2014; Perez-Martin et al., 2014), achieving good protection 1 day after treatment. However, for the prevention and treatment of FMD, a combined therapeutic approach involving molecules that induce immediate protection and a good vaccine that requires 5–7 days to elicit significant levels of neutralizing antibodies, will be necessary (Quattrocchi et al., 2014). Therefore, it is imperative to use biotherapeutic candidates with sustained biological antiviral activity ideally lasting for at least 5 days, to protect the host until the vaccine induces sufficient adaptive immunity. Initial studies by Moraes et al. (2003) showed that partial protection using Ad5-poIFNα was achieved when pigs were challenged at 5- or 7-days post-treatment. These results highlighted the need of other IFN platforms with extended half-life that could protect until vaccine induced immunity reached protective levels. In recent years, protein PEGylation has evolved as a viable successful alternative to change protein pharmacokinetics, due to an increase in the protein half-life in the recipient (Dozier and Distefano, 2015). In particular, PEGylation of type I IFNs to control human pathogens such as Hepatitis C, Hepatitis B, VEE, and others has been described (Lukaszewski and Brooks, 2000; Hoofnagle and Seeff, 2006; Friedman, 2008). However, its use in the veterinary field has never been reported. In this manuscript, we demonstrate for the first time that PEGylated poIFNα is an effective treatment to block FMDV replication in a natural host. Furthermore, PEGylation considerably extended efficacy of treatment from 1 to at least 5 days, conferring 100% protection. This result makes PEGylated poIFNα a very appealing biotherapeutic candidate. Use of this molecule with FMD vaccines seems like the perfect therapeutic combination to achieve early full and long-lasting protection. In this regard, IFNα would not only be effective at blocking initial FMDV replication, as demonstrated in this manuscript, but would also be an effective adjuvant for the FMD vaccine, as it has been previously described (de Avila Botton et al., 2006; Su et al., 2013; Duan et al., 2020). Further studies using the PEGpoIFNα in combination with FMD vaccines to confirm this adjuvant effect are warranted.

Despite the successful use of our Ad5-poIFNα technology against FMD, we have never achieved this level of protection when animals were challenged by 5 dpt (Moraes et al., 2003). Consistently however, with our previous studies with Ad5-poIFNα, protection was directly correlated with detectable levels of a systemic antiviral response at the moment of challenge with FMDV. Interestingly, there was not a statistically significant difference between the levels of antiviral activity induced by PEGpoIFNα detected at 1 or at 5 days post-treatment, confirmed by two methods of detection (MxCAT ELISA and conventional biological assay). These results indicated that PEGpoIFNα consistently elicited a strong and sustained biological activity in pigs.

IFN stimulates the transcriptional up-regulation of hundreds of effector genes (Takaoka and Yanai, 2006). We have previously described that swine treated with type I IFN delivered by the Ad5-vector platform induced the expression of ISGs systemically and locally in different tissues of relevance for FMDV infection (Diaz-San Segundo et al., 2010). To corroborate that treatment with PEGpoIFNα showed similar systemic gene upregulation we analyzed the expression of some well-characterized ISGs in PBMCs isolated from the treated pigs. Analysis of the relative levels of ISGs at the moment of challenge (1 and 5 dpt), showed that PEGpoIFNα induced upregulation of nine out of 15 analyzed genes by 1 dpt. Interestingly, by 5 dpt the number of upregulated genes and the fold induction increased considerably. Presumably this was the result of the sustained and cumulative effect induced by PEGpoIFNα, a stabilized long-acting molecule, as shown in the included PK data, and in previous studies with PEGylated human IFNs (Fried et al., 2002; Woo et al., 2017). Our results indicated that PEGylation of IFN did not affect its normal ability to bind its specific receptor and induce a systemic antiviral state. Three of the upregulated genes, PKR, OAS1, and CXCL10, have been directly involved in IFNα-induced antiviral effects against FMDV, since IFN treatment of PKR^–/–^ and OAS^–/–^ embryonic fibroblast or CXCL10^–/–^ mice could not block FMDV replication (Chinsangaram et al., 2001; de Los Santos et al., 2006; Diaz-San Segundo et al., 2013). CXCL10 and other chemokines like CCL2, CCL20, and CCR2 are involved in migration of immune cells (Charbonnier et al., 1999; Fujita et al., 2005). In fact, after Ad5-poIFNα treatment there is an increase in dendritic cells (DCs) in the skin and natural killer (NK) cells in the draining lymph-nodes, with partial maturation of DCs (Diaz-San Segundo et al., 2010, 2013). The antiviral state often induced by viral infection is mediated by cytoplasmic rig-I like receptors (RLRs). For picornaviruses like FMDV, with a positive-sense RNA genome, replication intermediates are predominantly recognized by mda-5 (Husser et al., 2011; Wang et al., 2011; Pulido et al., 2020). PEGpoIFNα not only induced mda-5 but also rig-I. Two other genes that are part of the cellular ISGylation machinery, the ubiquitin-like protein modifier ISG15 and the ubiquitin specific peptidase USP18, were also upregulated in response to PEGpoIFNα. It has recently been demonstrated that FMDV actively induces protein deISGylation (Medina et al., 2020a; Visser et al., 2020). Furthermore, overexpression of ISG15 and the ISGylation machinery results in moderate inhibition of FMDV replication in porcine cells (Medina et al., 2020a). These results suggested that ISG15 plays a critical role in controlling infection. On the other hand, two out of four animals in the control group showed upregulation of ISG15, suggesting that by itself this gene might not be sufficient to protect against FMDV challenge. Intricate interactions among several ISGs may be necessary to fully control FMD in the animal host. Future studies to evaluate gene expression but also the proteome of IFN induced responses in the specific animal hosts are necessary to elucidate the mechanisms by which IFN controls FMDV replication *in vivo*.

Overall, our results demonstrate that treatment with PEGpoIFNα induces a sustained strong and systemic antiviral state able to protect animals against challenge with FMDV for at least 5 days post-administration. Use of a recombinant purified pegylated protein might represent a more cost-effective alternative compared to protein-expression platforms delivered by live vectors such as Ad5. Ultimately, a combination of PEGpoIFNα with a FMD vaccine should provide early and long-lasting protection against a viral disease that spreads very rapidly during an outbreak and currently causes devasting consequences in vast areas of the World. Future experiments to confirm the efficacy of this combination treatment, to evaluate similar molecules that could be more effective in other FMDV host such as cattle, and to understand in more detail the mechanisms of protection in each susceptible species are warranted.

## Data Availability Statement

The raw data supporting the conclusions of this article will be made available by the authors, without undue reservation.

## Ethics Statement

The animal study was reviewed and approved by Institutional Animal Care and Use Committee (IACUC) of the Plum Island Animal Disease Center (USDA/APHIS/AC Certificate number: 21-F-0001; Protocol 151-19R).

## Author Contributions

FDSS conceived, performed experiments, analyzed data, wrote, and submitted the manuscript. GM performed experiments and analyzed the data. AM, PA, SA, and JG performed experiments. KL coordinated the pharmacokinetics study. JS performed the statistical design and analysis. TH managed the project at Animal Health, Inc. TS conceived, directed, and wrote the manuscript. All authors discussed design and results and contributed to the final writing and revisions of the manuscript.

## Conflict of Interest

The authors declare that this study was partially funded by Elanco Animal Health, Inc. The study was conceived and led by FDSS and TDLS who together with Elanco Animal Health discussed the design, analysis and interpretation of results, and wrote and submitted the manuscript for publication. KL, JS, and TH are employees at Elanco Animal Health, Inc. The remaining authors declare that the research was conducted in the absence of any commercial or financial relationships that could be construed as a potential conflict of interest.
